# Identification of a ferroptosis-related gene signature (FRGS) for predicting clinical outcome in lung adenocarcinoma

**DOI:** 10.7717/peerj.11233

**Published:** 2021-04-13

**Authors:** Sheng Wang, Chunlei Wu, Dehua Ma, Quanteng Hu

**Affiliations:** 1 Respiratory Department, Jinhua Guangfu Hospital, Jinhua, Zhejiang, China; 2 Department of Thoracic Surgery, Taizhou Hospital, Taizhou, Zhejiang, China

**Keywords:** Lung adenocarcinoma, Ferroptosis, Signature, Immune

## Abstract

**Background:**

Lung adenocarcinoma (LUAD) is the most common pathological subtype of lung cancer. Ferroptosis, an oxidative, iron-dependent form of necrotic cell death, is highly associated with tumorigenesis and cancer progression. However, the prognostic value of ferroptosis progress in LUAD was still rarely be investigated.

**Methods:**

Herein, we collected three mRNA expression profiles and 85 ferroptosis-related genes from public databases. The “limma” package was used to identify ferroptosis-related differentially expressed genes (DEGs). Univariate Cox regression analysis and LASSO regression analysis were applied to screen and develop a ferroptosis-related gene signature (FRGS) and a formula to calculate the risk score. Multivariate Cox regression analysis was implemented to determine independent prognostic predictors of overall survival (OS). The area under the receiver operating characteristic curve (AUC) and calibration plot were used to evaluate the predictive accuracy of the FRGS and nomogram.

**Results:**

We developed a FRGS with five genes (CYBB, CISD1, FADD, SAT2, VDAC2). The AUC of the FRGS in TCGA cohort was 0.777 at 1-year, 0.721 at 3-year and 0.725 at 5-year, significantly superior to the AUC of TNM stage (1-year: 0.701, 3-year: 0.691, 5-year: 0.686). A similar phenomenon was observed in GEO cohort 1 and 2. Multivariate Cox regression analysis indicted TNM stage and risk score were independent prognostic predictors. Finally, we built a nomogram with TNM stage and FRGS, the AUCs of which markedly higher than that of FRGS or TNM stage alone.

**Conclusion:**

We constructed a prognostic FRGS with five ferroptosis-related genes and a nomogram for predicting the 1-, 3- and 5-year survival rate of LUAD patients, which may provide a new understanding of the prognostic value of ferroptosis progress in LUAD and will benefit prognosis assessment of LUAD patients.

## Introduction

Lung cancer (LC), the highest incidence rate of malignancy globally, is estimated that nearly 234,000 new cases are diagnosed per year, and accounts for 13% and 14% new cancer cases in women and men, respectively (Bray et al., 2018; De Groot et al., 2018). Non-small cell lung cancer (NSCLC) is the most common subtype of LC (85%), and lung adenocarcinoma (LUAD) is the leading histology of NSCLC and accounts for over 65% of NSCLC cases (De Groot et al., 2018; Yu, Zhang & Zhang, 2020). With the advance of treatment strategies such as targeted therapy and immunotherapy, the long term survival rate of LUAD patients is continuously increasing. However, the five-year survival of LC patients at stage I remains poor at 65%, and for advanced patients is still less than 20% (Goldstraw et al., 2007; Herbst, Morgensztern & Boshoff, 2018). Thus, there is an urgent need to identify prognostic biomarkers and develop an effective prognostic model for predicting the prognosis of LUAD, which may be conducive to risk stratification and clinical treatment of LUAD.

Based on morphotype, cell death is classified into three types: apoptosis, autophagy and necrosis. In recent years, some novel discovered cell death processes have altered our traditional understanding of cell death (Liang et al., 2019; Proneth & Conrad, 2019). Among them, ferroptosis has gained considerable attention from researchers. Unlike autophagy and apoptosis, ferroptosis is defined as an oxidative, iron-dependent form of necrotic cell death, characterized by the generation of lethal amounts of lipid peroxidation products and reactive oxygen species (ROS) (Proneth & Conrad, 2019). Emerging evidence showed that some oncogenic pathways were related to ferroptosis and eradicate the carcinogenic cells by adjusting ferroptosis (Mou et al., 2019; Hassannia, Vandenabeele & Vanden Berghe, 2019). For example, P53, a well-studied tumor suppressor gene, could repress the expression of cystine/glutamate antiporter at the transcriptional level to regulate the ferroptosis pathway (Xie et al., 2017). In addition, clues demonstrated that extra-mitochondrial lipid peroxidation arising from an iron-dependent ROS accretion could trigger ferroptosis to inhibit tumorigenesis (Liang et al., 2019). Nevertheless, as reported in previous studies, ferroptosis process in lung tissues is generally inhibited due to the up-regulation of cystine/glutamate antiporter and iron reduction, which contribute to the tumorigenesis and development of LC (Lai et al., 2019). Whether ferroptosis process and relevant ferroptosis-related genes are correlated with the clinical outcomes of LUAD remains to be further elucidated.

In the present study, we collected ferroptosis-related genes and mRNA expression profiles to construct a novel ferroptosis-related gene signature (FRGS) and nomogram for predicting overall survival (OS) of LUAD patients, which may be help to understand the potential prognostic value of ferroptosis-related genes in LUAD, and provide a convenient tool for risk assessment and prognosis assessment in LUAD.

## Materials and Methods

### Data collection

Three independent cohorts were collected in this study, including TCGA cohort, GEO cohort 1 and GEO cohort 2. The level 3 RNA sequencing of TCGA cohort was retrieved from TCGA database (TCGA-LUAD, The Cancer Genome Altas, https://www.cancer.gov). The raw data of mRNA expression matrix of GSE68465 (GEO cohort 1) and GSE41271 (GEO cohort 1) were gathered from GEO database (Gene Expression Omnibus, https://www.ncbi.nlm.nih.gov). The platform of GSE68465 was GPL96 (Affymetrix Human Genome U133A Array), and for GSE41271, was GPL6884 (Illumina HumanWG-6 v3.0 expression beadchip), GSE68465 was normalized with the MAS5.0 method using the “affy” package, and GSE41271 was normalized with the Bioconductor package “lumi”. In addition, corresponding clinical information of all patients were also collected, such as age, gender, smoking, TNM stage, overall survival time (OS), and survival status. Cases lacking survival time and survival status would be removed. Ferroptosis-related genes were obtained from KEGG Pathway Database (https://www.genome.jp/dbget-bin/www_bget?pathway:map04216), and previous literature (Mou et al., 2019; Stockwell et al., 2017; Doll et al., 2019).

### Construction and evaluation of FRGS

In TCGA cohort, the “limma” package in R was used to identify ferroptosis-related differentially expressed genes (DEGs) between normal and LUAD tissues. |log2FC| > 1 and false discovery rate (FDR) < 0.01 were set as the criteria. Then, DEGs were subjected to univariate Cox regression analysis to screen out OS-related genes using *P*-value < 0.05 as the cut-off criterion. Finally, LASSO regression analysis was applied to further filtrate prognostic candidate ferroptosis-related DEGs, and construct a FRGS and ferroptosis-related risk score formula with the “glmnet” R package.

$$Risk\;score=\overset{n}{\underset{i=1}{\sum }}coeffcient*Expression\;of\;FRG\left(i\right)$$

Based on this formula, we calculated the risk score of each patient, and divided patients into low- and high- risk score groups according to the optimal cut-off value of risk score determined with R package “Survminer”. The principal component analysis (PCA) was performed to investigate the expression difference between low-and high-risk groups. The receiver operating characteristic curve (ROC) was depicted and the area under the curve (AUC) was calculated to evaluate the predictive power of the FRGS. Additionally, with a boot-strapping set of 1,000 resamples, the calibration plot was carried out to assess the FRGS. In the same way, the risk score of each patient was calculated, and the ROC and calibration plot were also performed in GEO cohort 1 and 2 to validate the predictive effectiveness of the FRGS.

### Gene set enrichment analysis (GSEA)

To determine the potential altered signaling pathways between low- and high- risk patients, GSEA was performed with FDR < 0.05 as the threshold.

### Single-sample gene set enrichment analysis (ssGSEA)

The ssGSEA was performed to assess the level of immune cells and the activity of immune-related pathways with BiocManager package: GSVA. The method for ssGSEA was based on a rank value of each gene, which defined a score representing the degree of absolute enrichment of a particular gene set in each sample. 29 gene sets specific (16 immune cell gene sets and 13 immune-related pathway sets) were acquired from other studies (Charoentong et al., 2017; Shi et al., 2020; Zuo et al., 2020).

### Statistical analysis

All statistical analyses were performed with R 3.6.3 (https://www.r-project.org). For categorical data, group comparison was conducted with chi-square test, and for measurement data, was with *t*-test or one-way ANOVA. The Spearman correlation test analyzes the correlation among candidate ferroptosis-related candidate DEGs. Kaplan–Meier plot was carried out to investigate the relation of OS to the ferroptosis-related risk score as well as the expression of ferroptosis-related candidate DEGs. Univariate Cox regression analysis and multivariate Cox regression analysis were implemented to determine independent prognostic predictors of OS.

## Results

### Patient cohort

**Figure 1:**
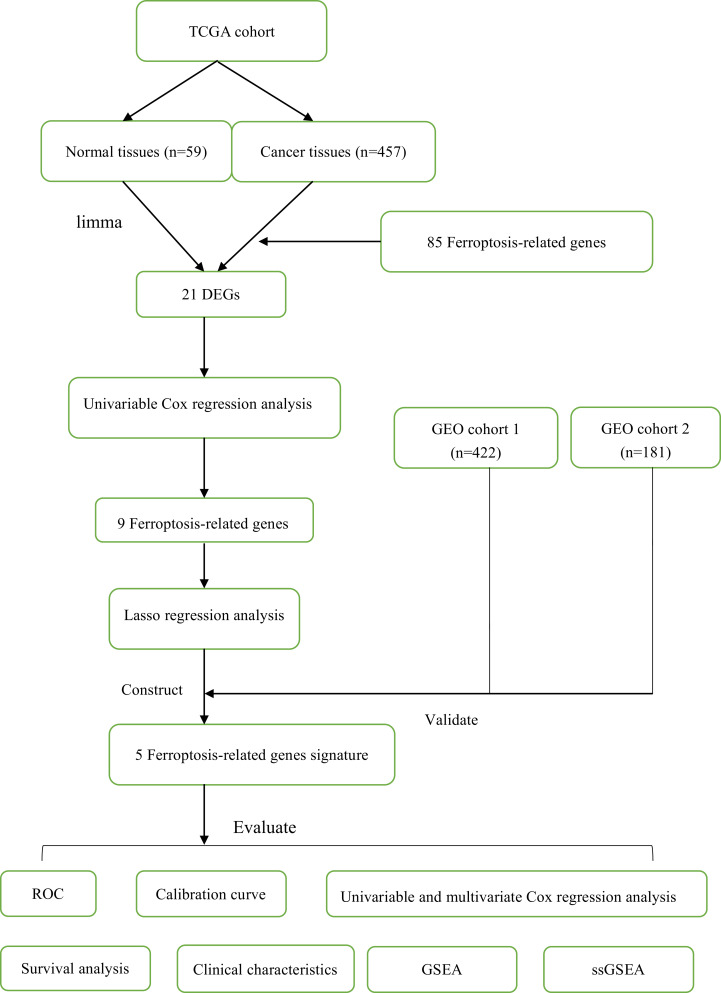
The flow diagram of this study.

In this study, a total of 1060 LUAD patients were enrolled from three independent cohorts (TCGA cohort: 457; GEO cohort 1: 422; GEO cohort 2: 181). The detailed demographic and baseline characteristics of all samples were presented in Table 1. Besides, we obtained 85 ferroptosis-related genes. The flow diagram of this study was shown in Fig. 1.

**Table 1:** The baseline characteristics of lung adenocarcinoma patients in this study.

Parameter	TCGA cohort	GEO cohort 1	GEO cohort 2
Database	TCGA	GSE68465	GSE41271
Gender			
Female	251 (54.92%)	211 (50.00%)	90 (49.72%)
Male	206 (45.08%)	211 (50.00%)	91 (50.28%%)
Age			
≤65	233 (50.98%)	219 (51.90%)	102 (56.35%)
>65	224 (49.02%)	203 (48.10%)	79 (43.65%%)
Smoking			
Never	61 (13.35%)	48 (11.37%)	26 (14.36%)
Ever	379 (82.93%)	288 (68.25%)	155 (85.64%%)
NA	12 (2.62%)	86 (20.38%)	0
TNM stage			
I	262 (57.33%)	267 (63.27%)	100 (55.25%)
II	102 (22.32%)	95 (22.51%)	28 (15.47%)
III	70 (15.32%)	60 (14.22%)	49 (27.07%)
IV	23 (5.03%)	0	4 (2.21%)
Tumor size			
T1	149 (32.60%)	146 (34.60%)	NA
T2	241 (52.74%)	238 (56.40%)	NA
T3	49 (10.72%)	27 (6.40%)	NA
T4	18 (3.93%)	11 (2.60%)	NA
NA	0	0	181 (100%)
Lymph node			
N0	311 (68.05%)	289 (68.48%)	NA
N1-3	146 (31.95%)	133 (31.52%)	NA
NA	0	0	181 (100%)
Metastasis			
M0	434 (94.97%)	422 (100%)	NA
M1	23 (5.03%)	0	NA
NA	0	0	181 (100%)
Survival status			
Alive	306 (66.96%)	193 (45.73%)	112 (61.88%%)
Dead	151 (33.04%)	229 (54.27%%)	69 (38.12%)
Risk score			
Low	272 (59.52%)	89 (21.09%)	122 (67.40%)
High	185 (40.48%)	333 (78.91%)	59 (32.60%)
Total	457 (100%)	422 (100%)	181 (100%)

**Note:**

TCGA, The Cancer Genome Altas; GEO, Gene Expression Omnibus; NA, represents information not available.

### Construction, validation and evaluation of the prognostic FRGS

**Figure 2:**
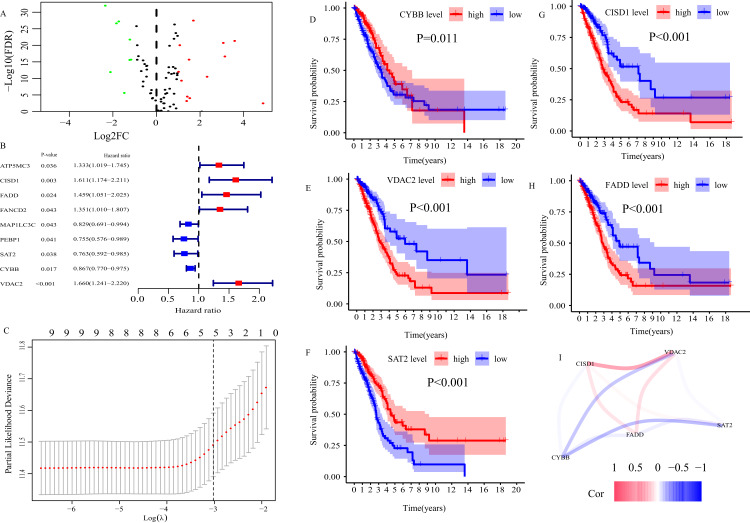
Construction of ferroptosis-related gene signature (FRGS) in TCGA cohort. (A) Volcano plot of ferroptosis-related DEGs between normal and LUAD tissues with |log2FC| > 1 and FDR < 0.01. Red dots represent 13 up-regulated genes and green dots represent eight down-regulated genes. (B) Univariate Cox regression analysis identified nine ferroptosis-related genes were related to overall survival (P < 0.05). (C) “Leave- one-out-cross-validation” for parameter selection in LASSO regression to filter out five candidate genes. (D) Survival analysis of CYBB, (E) CISD1, (F) VDAC2, (G) FADD, (H) SAT2. (I) The correlation network of five candidate genes. DEG, differentially expressed genes; FDR, false discovery rate.

In TCGA cohort, 21 ferroptosis-related DEGs between normal and LUAD tissues were determined (Fig. 2A). Then, 21 DEGs were subjected to univariate Cox regression analysis, and 9 genes highly related to OS of LUAD patients were screened out (*P* < 0.05, Fig. 2B). Finally, we applied LASSO regression analysis to analyze those 9 genes, and constructed a prognostic FRGS with 5 candidate ferroptosis-related genes (CYBB, CISD1, FADD, SAT2, VDAC2, Fig. 2C). Survival analysis showed that high-level CYBB (*P* = 0.011, Fig. 2D) and SAT2 (*P* < 0.001, Fig. 2F) along with low-level CISD1 (*P* < 0.001, Fig. 2G), FADD (*P* < 0.001, Fig. 2H) and VDAC2 (*P* < 0.001, Fig. 2E) predicted better favorable outcomes in LUAD patients. The correlation among 5 candidate ferroptosis-related genes is presented in Fig. 2I. The risk score formula was presented as follows.

$$\begin{array}{cc} Risk\;score\; & =\;\left(0.608*\exp of\;CISD1\right)\;+\;\left(-0.093*\exp of\;CYBB\right)\;\\ & +\;\left(0.429*\exp of\;FADD\right)\;+\;\left(-0.326*\exp of\;SAT2\right)\;\\ & +\;\left(0.230*\exp of\;VDAC2\right)\; \end{array}$$

**Figure 3:**
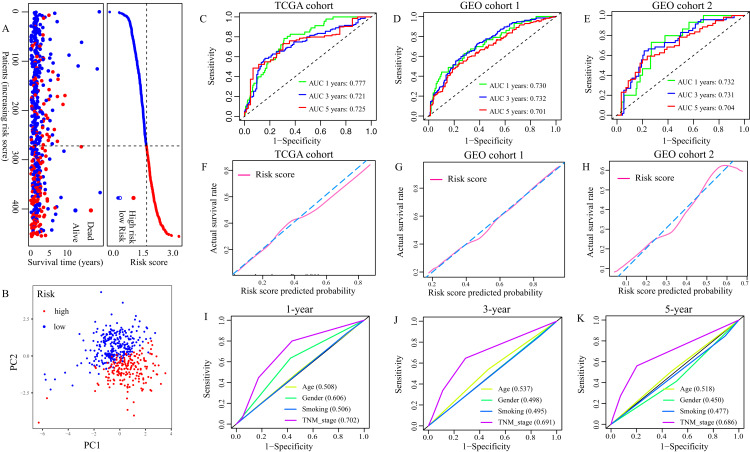
Evaluation of the FRGS. (A) The distribution of risk score and survival status in TCGA cohort. (B) PCA showed that the patients in different risk groups were gathered in two areas. (C–E) The ROC of the FRGS for predicting 1-, 3- and 5-year OS in TCGA cohort, GEO cohort 1, and GEO cohort 2. (F–H) The calibration plots of TCGA cohort, GEO cohort 1 and GEO cohort 2. (I–K) The ROC of clinical indexes for predicting 1-, 3- and 5-year OS in TCGA cohort. PCA, principal component analysis; ROC, the receiver operating characteristic curve.

According to the formula, the risk score of each patient in TCGA cohort was calculated, and patients were divided into low- and high-risk groups based on the optimal cut-off risk score:1.515 (Fig. S1A). So did patients in GEO cohort 1 and 2. The distributions of risk score and survival status in TCGA cohort were shown in Fig. 3A. PCA of TCGA cohort demonstrated that the patients in different risk groups were gathered in two areas (Fig. 3B), which was confirmed in GEO cohort 1 and 2 (Figs. S1B and S1C).

Then, we depicted the ROC of the FRGS, and estimated the value of AUC at 1-, 3- and 5- year. The AUC of the FRGS in TCGA cohort was 0.777 at 1-year, 0.721 at 3-year and 0.725 at 5-year (Fig. 3C), higher than that of clinical indexes (Figs. 3I–3K). To confirm the accuracy of the survival probabilities of the FRGS, we validate the finding in GEO cohort 1 with AUCs at 1-, 3- and 5-year OS reaching 0.730, 0.732 and 0.701, respectively (Fig. 3D), and in GEO cohort 2 with AUCs at 1-, 3- and 5-year OS reaching 0.732, 0.731 and 0.704, respectively (Fig. 3E). Similarly, the AUCs in GEO cohort 1 and 2 were more excellent than that of clinical indexes (Fig. S2). The calibration plots of three cohorts were presented in Figs. 3F and 3H.

### Correlation between FRGS and clinical characteristics

**Figure 4:**
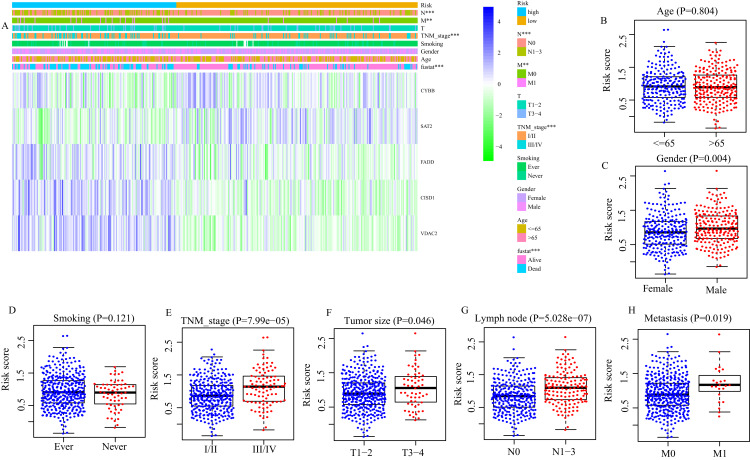
Correlation between risk score and clinical characteristics. (A) The distribution of clinicopathological features between high- and low- risk group. (B) The difference of risk score between Age ≤ 65 and Age > 65, (C) between different gender, (D) smoking and non-smoking, (E) TNM stage I/II and III/IV, (F) T1/2 and T3/4, (G) between with and without lymph node metastasis, (H) between with and without distance metastasis. **P < 0.01, ***P < 0.001.

**Figure 5:**
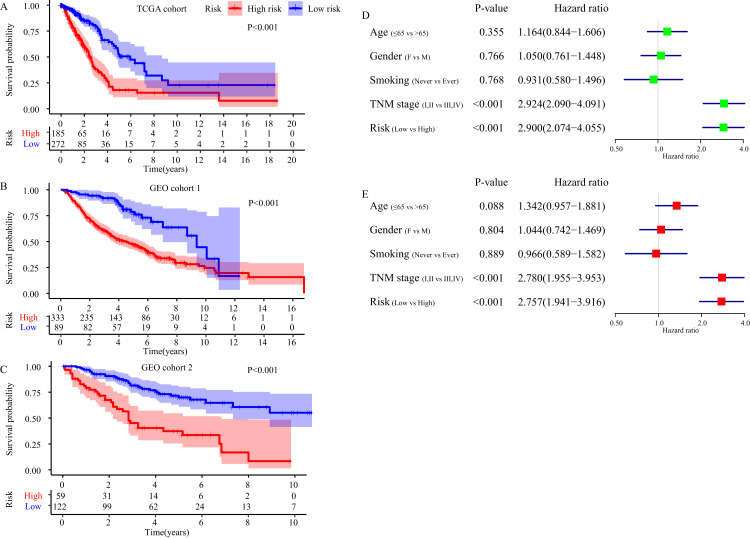
The FRGS is an independent prognostic factor for the prognosis of LUAD patients. (A–C) Survival difference between high- and low-risk group in TCGA cohort, GEO cohort 1 and GEO cohort 2. (D) Result of the univariate Cox regression analysis in TCGA cohort. (E) Results of the multivariate Cox regression analysis in TCGA cohort.

Next, we evaluated the correlation between FRGS and clinical characteristics (age, gender, smoking, TNM stage, tumor size, lymph node metastasis and distant metastasis). Between low- and high-risk groups, the distributions of patients at different TNM stage (*P* < 0.001), with or without lymph node metastasis (*P* < 0.001) and distant metastasis (*P* = 0.013) was significantly different (Fig. 4A). Moreover, the risk score in male patients was significantly increased (*P* = 0.004, Fig. 4C). Similar results were observed in patients staged III/IV (*P* < 0.001, Fig. 4E), Tumor size at T3/4 (*P* = 0.045, Fig. 4F), with lymph node metastasis (*P* < 0.001, Fig. 4G), and with distant metastasis (*P* = 0.019, Fig. 5H). However, we did not found the effect of age (*P* = 0.804, Fig. 4B) and smoking (*P* = 0.121, Fig. 4D) on the risk score.

### Independent prognostic value of the FRGS

**Figure 6:**
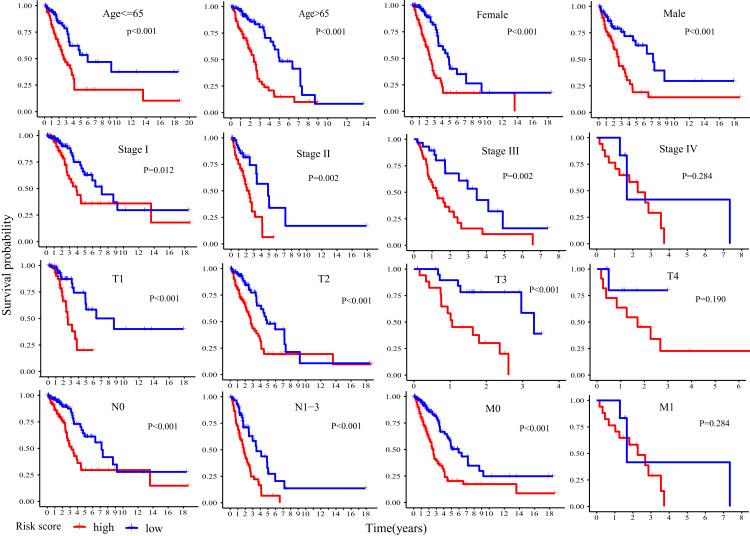
Stratification analyses of overall survival difference between high- and low- risk patients in different subgroups.

Kaplan-Meier plot was executed to explore overall survival difference between two risk groups, and the result in TCGA cohort demonstrated that the prognosis of high-risk patients was worse than that of low-risk patients (*P* < 0.001, Fig. 5A), in line with the results in GEO cohort 1 (*P* < 0.001, Fig. 5B) and GEO cohort 2 (*P* < 0.001, Fig. 5C). Furthermore, stratification analyses indicated high-risk patients exhibited poorer clinical outcomes in each subgroup except in subgroup Stage IV, T1 and M1 (Fig. 6).

Then, we took advantage of univariate and multivariate Cox regression model to compare the risk score with clinical parameters (age, gender, smoking, TNM stage) in TCGA cohort to determine whether the risk score was an independent prognostic predictor for OS of LUAD patients. Univariate Cox regression analysis showed that the risk score was an important OS-related influence factor (HR = 2.900, 95% CI [2.074–4.055], *P* < 0.001, Fig. 5D). We verified the result in GEO cohort 1 (HR = 2.820, 95% CI [1.831, 4.342], *P* < 0.001, Fig. S3A) and GEO cohort 2 (HR = 3.511, 95% CI [2.170–5.681], *P* < 0.001, Fig. S3C), and obtained the same results. In addition, multivariate Cox regression model in TCGA cohort indicated the risk score was an independent influence factor for OS of LUAD patients (HR = 2.757, 95% CI [1.941–3.916], *P* < 0.001, Fig. 5E). It was validated in GEO cohort 1 (HR = 2.418, 95% CI [1.543–3.789], *P* < 0.001, Fig. S3B) and GEO cohort 2 (HR = 3.390, 95% CI [2.062–5.574], *P* < 0.001, Fig. S3D).

### GSEA

**Figure 7:**
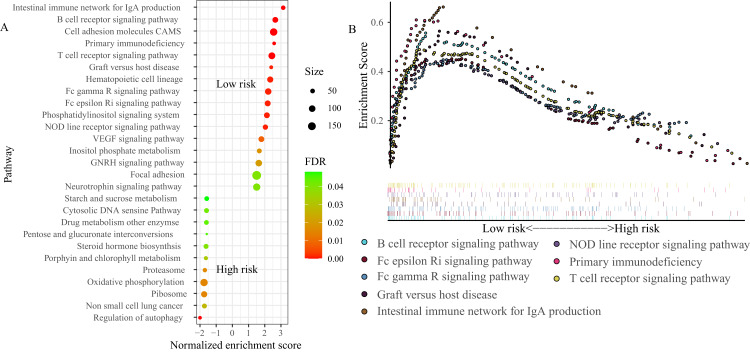
Gene set enrichment analysis (GSEA) between high- and low-risk groups. (A) A total of 27 pathways were identified, including 16 pathways in low-risk group and 11 pathways in high-risk group. (B) Eight immune-related pathways enriched in low-risk group. NES, normalized enrichment score; ES, enrichment score.

To explore the basic biological mechanisms of the FRGS, we performed GSEA analysis. A total of 27 pathways were identified, including 16 pathways in low-risk group and 11 pathways in high-risk group (Fig. 7A). Of note, several immune-related pathways were enriched in low-risk group, such as B cell receptor signaling pathway (Normalized enrichment score (NES): 2.72, FDR < 0.001), T cell receptor signaling pathway (NES: 2.52, FDR < 0.001), Intestinal immune network for IgA production (NES: 3.19, FDR < 0.001), NOD line receptor signaling pathway (NES: 2.1, FDR = 0.001), Fc epsilon Ri signaling pathway (NES: 2.25, FDR = 0.001), Fc gamma R signaling pathway (NES: 2.29, FDR = 0.001), and Graft versus host disease (NES: 2.44, FDR = 0.001, Fig. 7B).

### ssGSEA

**Figure 8:**
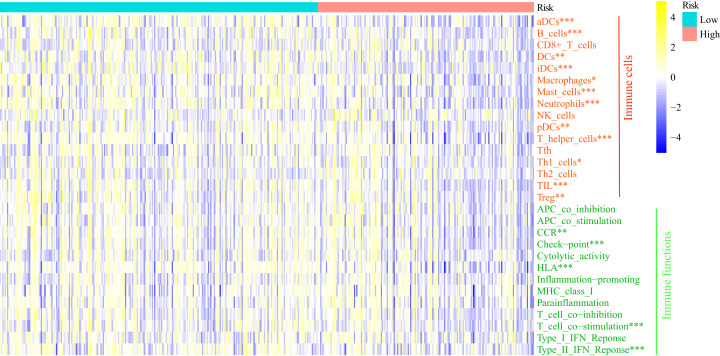
Comparison of the enrichment score of ssGSEA between different risk groups. aDCs, activated dendritic cells; DCs, dendritic cells; iDCs, immature dendritic cells; pDCs, plasmacytoid dendritic cells; Tfh, T follicular helper cells; Th1_cells, type 1 helper cells; Th2_cells, type 2 helper cells; TIL, tumor infiltrating lymphocytes; Treg, regulatory T cells; HLA, human leukocyte antigen; CCR: C–C chemokine receptor; APC, antigen presenting cells; MHC, major histocompatibility complex. *P < 0.05, **P < 0.01, ***P < 0.001.

GSEA analysis showed the FRGS was highly associated with immune status. Thus, we carried out ssGSEA to investigate the relationship of FRGS to the enrichment score of 16 immune cells and 13 immune-related pathways (Fig. 8). Interestingly, the enrichment score of aDCs, DCs, iDCs, pDCs, B cells, Macrophages, Mast cells, Neutrophils, T helper cells, Th1 cells, TIL and Treg was significantly increased in low-risk group. Meanwhile, low-risk group had a higher score of CCR, the activity of checkpoint molecules, HLA, T cell co−stimulation and IFN Reponse Type II.

### Construction, validation and evaluation of of nomogram

**Figure 9:**
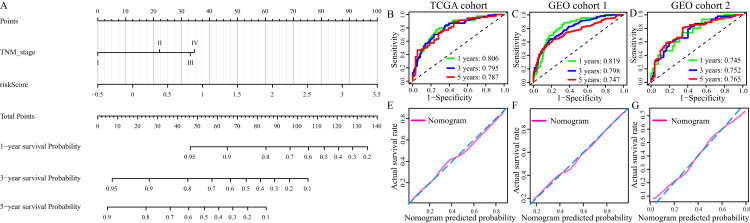
Construction and evaluation of the prognostic nomogram. (A) The prognostic nomogram for the prediction of 1-, 3- and 5-year overall survival in LUAD. (B–D) The ROC of the nomogram for predicting 1-, 3- and 5-year OS in TCGA cohort, GEO cohort 1, and GEO cohort 2. (E–G) The calibration plots of TCGA cohort, GEO cohort 1 and GEO cohort 2.

Multivariate Cox regression analysis identified TNM stage and risk score were independent OS-related predictors. Therefore, we used TNM stage and risk score to develop a prognostic nomogram in TCGA cohort (Fig. 9A). The AUCs of the nomogram for predicting 1-, 3- and 5- year OS in TCGA cohort were 0.806, 795 and 0.787, respectively (Fig. 8B). Then, we validated the predictive power of the nomogram in GEO cohort 1 and 2. The AUCs in GEO cohort 1 reached 0.819 at 1-year, 0.798 at 3-year and 0.747 at 5-year, and in GEO cohort 2 reached 0.745 at 1-year, 0.752 at 3-year and 0.765 at 5-year (Figs. 8C and 8D). The calibration plots of the nomogram in three cohorts were presented in Figs. 8E and 8F.

## Discussion

In recent years, due to air pollution, smoking and other factors, the incidence rate of LC is continuously rising. And, LUAD has become the most common histological subtype of LC (Bray et al., 2018; De Groot et al., 2018). Although, increasingly advanced diagnosis and treatment strategy greatly improved the long term survival rate of LUAD patients. The five-year survival rate remains less than 20% (Herbst, Morgensztern & Boshoff, 2018). TNM stage (AJCC) is still the most commonly used parameter for clinical decision and prognosis evaluation in LUAD. However, emerging clinical evidences show that patients with the same TNM stage and treatment strategy have different prognoses, indicating that prognosis assessment based on TNM stage alone may be not adequate in LC.

Ferroptosis, as an oxidative, iron-dependent form of necrotic cell death, has attracted a lot of attention. Researches realized that several oncogenic pathways render tumor cells extremely susceptible to ferroptosis through regulating key checkpoints, which could promote cancer cells death and repress tumor progression (Mou et al., 2019; Hassannia, Vandenabeele & Vanden Berghe, 2019) Previous studies showed modulation of ferroptosis progress could alter proliferation, colony formation, and cell death of LC cells and improve the therapeutic effect on xenograft of LC (Alvarez et al., 2017; Wang et al., 2019a; Gai et al., 2020). However, the role of ferroptosis on LUAD patients’ OS remains largely unknown.

In the current study, we collected 85 ferroptosis-related genes and three mRNA expression matrixes of LUAD patients (TCGA-LUAD, GSE68465 and GSE41271). In TCGA cohort, 21 ferroptosis-related DEGs were identified between normal and LUAD tissues. After screened by univariate Cox regression analysis and LASSO regression analysis, 5 ferroptosis-related genes (CYBB, CISD1, FADD, SAT2, VDAC2) were applied to construct a novel ferroptosis-related gene signature (FRGS). Survival analysis illustrated that all of those 5 genes were highly related to the OS of LUAD. FADD (Fas Associated Via Death Domain) is an adaptor molecule that, through recruiting caspase-8 or caspase-10, could activate Fas (CD95) or TNFR-1 receptors to mediate cell death signals. In addition, it is also involved in cell cycle progression, T-cell proliferation, and interferon-mediated immune response (Marín-Rubio et al., 2019). Previous researches have proven that overexpression of FADD predicted unfavorable clinical outcomes in NSCLC (Bhojani et al., 2005; Chen et al., 2005; Chen et al., 2020), which was in line with the finding of this study. Experimental studies showed that NSCLC cells could release FADD which was positively related to the progression and aggressiveness of LC and phosphorylated FADD can induce cell-cycle progression and cell proliferation in LC (Chen et al., 2005; Chen et al., 2020). VDAC2 (Voltage Dependent Anion Channel 2) encodes a protein that participates in metabolite diffusion across the mitochondrial outer membrane and mitochondrial apoptotic pathway (Chin et al., 2018). A basic study about melanoma demonstrated VDAC2 could suppress ferroptosis (Yang et al., 2020). Additionally, Wang et al. found increased VDAC2 protected pancreatic cancer cells from chemotherapy via restraining apoptosis (Chin et al., 2018). The protein encoded by CISD1 (CDGSH Iron Sulfur Domain 1) localizes to the outer membrane of mitochondria, and binds to a redox-active [2Fe-2S] cluster (Taminelli et al., 2008; Yuan et al., 2016). It plays an important role in the regulation of oxidation and iron metabolism. And, CISD1 could limit mitochondrial iron uptake and therefore inhibit ferroptosis (Taminelli et al., 2008). CYBB (Cytochrome B-245 Beta Chain) is a primary component of the oxidase system of phagocytes, and is involved in the killing effect of multiple immune cells (Monteiro et al., 2013). SAT2 (Spermidine/Spermine N1-Acetyltransferase Family Member 2) could catalyze the N-acetylation of the amino acid thialysine, and enhance NF-kappaB-dependent transcription (Vogel, Boeke & Ashburner, 2006). Although, many previous studies have reported the biological functions of those 5 ferroptosis-related genes. Up to now, little is known regarding the role of those genes on LUAD especially CYBB, CISD1, SAT2 and VDAC2. Hence, it is needed more basic and clinical researches to further clarify the biological functions of those 5 ferroptosis-related genes in LUAD.

Herein, we developed and validated a FRGS for predicting OS of LUAD patients. Survival analysis demonstrated that high-risk patients had poorer clinical outcomes. And stratification analyses showed the same results in each subgroup except in subgroup Stage IV, T1 and M1, which may be because of insufficient sample size. The AUCs of the FRGS in TCGA cohort were 0.777 at 1-year, 0.721 at 3-year and 0.725 at 5-year, significantly superior to the AUCs of TNM stage (1-year: 0.701, 3-year: 0.691, 5-year: 0.686). A similar phenomenon was observed in GEO cohort 1 and 2. Additionally, the calibration plots demonstrated a good fit between the predicted probability and the actual survival rate. Multivariate Cox regression analysis identified TNM stage and risk score were independent factors for OS. Therefore, we applied those two parameters to construct a novel prognostic nomogram for estimating 1-, 3- and 5-year survival rate of LUAD patients. The AUCs of the nomogram in three cohorts were similar, and markedly higher than that of FRGS or TNM stage alone, indicating the nomogram is stable and reliable for predicting 1-, 3- and 5-year survival rate of LUAD. Moreover, the calibration plots showed better consistency than that of FRGS along. In recent years, several prognostic signatures based on RNA-seq or microarray expression have been established for exploring prognosis-related biomarkers and predicting OS of LUAD. For example, a previous study developed an autophagy-related gene prognostic signature with the AUC = 0.615 at 5-year (Zhu, Wang & Hu, 2020). And, an immune signature for 1- and 3-year survival rate of LUAD with AUCs reaching 0.70 and 0.68 (Guo et al., 2020). Both the AUCs of them were inferior to that of the nomogram. All data suggested that the established prognostic nomogram is suitable for predicting the 1-, 3- and 5-year survival probability of LUAD patients. Previously, Gao et al. established a prognostic signature with 12 ferroptosis genes (Gao et al., 2021). Those 12 ferroptosis genes were totally different with the ferroptosis-related genes in the study, which may be caused by different way used in developing signature. Gao et al. only applied LASSO regression analysis to screen prognostic candidate ferroptosis-related genes, and construct a signature. Herein, we firstly used univariate Cox regression analysis to filter out OS-related genes. Then, we applied LASSO regression analysis to further filtrate prognostic ferroptosis-related genes, and develop a FRGS. In addition, we collected more ferroptosis-related genes, and patient cohorts.

Recently, it has been realized that ferroptosis is involved in tumor immunization and cancer immunotherapy. In the present study, we performed GSEA and ssGSEA to investigate the basic biological mechanisms of the FRGS, and the relation of the FRGS to immune system. GSEA analysis demonstrated several immune-related pathways were enriched, especially in low-risk group. Meanwhile, ssGSEA results showed the enrichment score of multiple immune cells and immune-related pathways in low-risk group were increased, hinting that the patients with low-risk score may have better immune status and immune function. Wang et al. reported that CD8+ T cells could drive ferroptosis in tumor cells, and the immune system can regulate the sensitization of tumor cells to ferroptosis to suppress tumorigenesis (Wang et al., 2019b). In addition, immunotherapy enhanced tumor lipid oxidation and ferroptosis by repressing SLC7A11 to improve tumor control (Lang et al., 2019). All of these evidences highlighted the promising role of ferroptosis in cancer therapy, and targeting tumor ferroptosis pathway may be a new therapeutic way in combination with immunotherapy. Yet, undeniably, more work is warranted to explore the immunomodulatory role of ferroptosis in anti-tumor immunity.

Although the prognostic FRGS and nomogram presented a well predictive accuracy and effectiveness for OS of LUAD patients in this study, there were still some limitations that needed to be addressed. Firstly, it was a retrospective study, and all cases were retrospective samples. Hence, validation of prospective samples was still needed. Secondly, owing to all samples were collected from public databases, the potential selection bias could not be excluded. Thirdly, the signature was constructed based on microarray expression and RNA-seq data, which is costly and time-consuming. And, it lacked validation using PCR or IHC. Hence, further investigation is demanded to examine the discovery of this research both in vitro and in vivo.

Together, in this study, we constructed a prognostic FRGS with 5 ferroptosis-related genes (CYBB, CISD1, FADD, SAT2, VDAC2), and a nomogram for predicting the 1-, 3- and 5-year survival rate of LUAD patients. Evidences indicated that the nomogram was stable and reliable for the prediction of LUAD prognosis. Our study provides a new understanding of the prognostic value of ferroptosis progress in LUAD and will benefit the prognosis assessment of LUAD patients. However, the underlying mechanisms of ferroptosis-related genes on LUAD, and its relation to tumor immune status remain relatively enigmatic and warrant further investigation.

## Supplemental Information

10.7717/peerj.11233/supp-1 Supplemental Information 1 Determination and distribution of high- and low-risk group. (A) Determination of the optimal cut-off risk score:1.515. (B) PCA showed that the patients in different risk groups were gathered in two areas in GEO cohort 1. (C) PCA showed that the patients in different risk groups were gathered in two areas in GEO cohort 2.

10.7717/peerj.11233/supp-2 Supplemental Information 2 The ROC of clinical indexes for predicting 1-, 3-, and 5-year OS in GEO cohort 1 and 2.

10.7717/peerj.11233/supp-3 Supplemental Information 3 Verification of the FRGS as an independent prognostic factor for the prognosis of LUAD patients. (A) Result of the univariate Cox regression analysis in GEO cohort 1. (B) Results of the multivariate Cox regression analysis in GEO cohort 1. (C) Result of the univariate Cox regression analysis in GEO cohort 2. (D) Results of the multivariate Cox regression analysis in GEO cohort 2.

## Abbreviations

LC: Lung cancer
NSCLC: Non-small cell lung cancer
LU: AD lung adenocarcinoma
DEGs: differentially expressed genes
FRG: S ferroptosis-related gene signature
TC: GA The Cancer Genome Altas
GE: O Gene Expression Omnibus
RO: C the receiver operating characteristic curve
AU: C the area under curve of ROC
O: S overall survival
G: SEA Gene set enrichment analysis
N: ES Normalized enrichment score
s: sGSEA Single-sample gene set enrichment analysis
F: DR false discovery rate

## Additional Information and Declarations

### Data Availability

The following information was supplied regarding data availability: All data came from the TCGA dataset (TCGA-LUAD, The Cancer Genome Altas, https://www.cancer.gov) and the GEO database (GSE68465, GSE41271).
