# Supplementary figures and images for: Identification of a ferroptosis-related gene signature (FRGS) for predicting clinical outcome in lung adenocarcinoma
Source: PeerJ. 2021 Apr 13;9:e11233. doi: 10.7717/peerj.11233 (PMC8051350; doi:10.7717/peerj.11233)

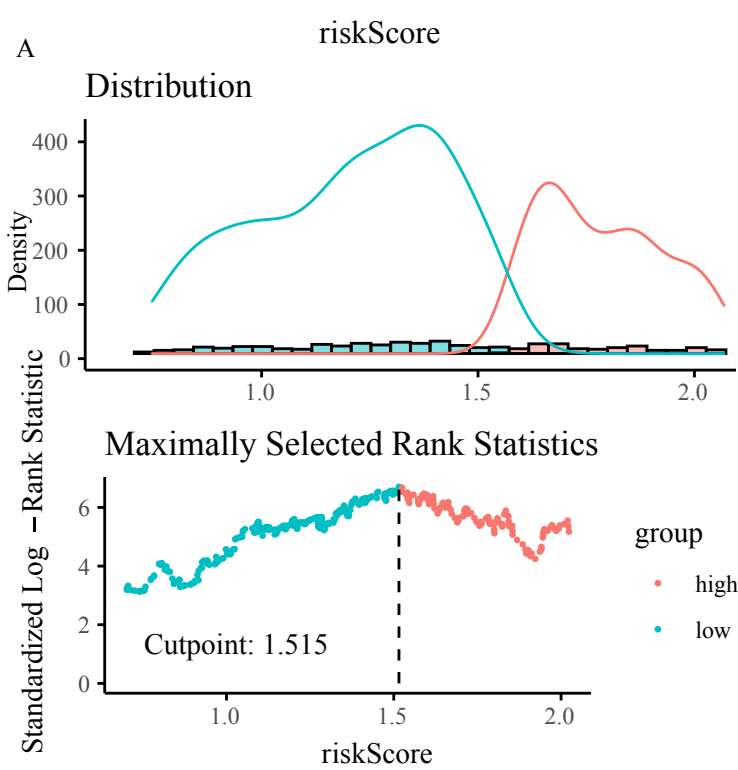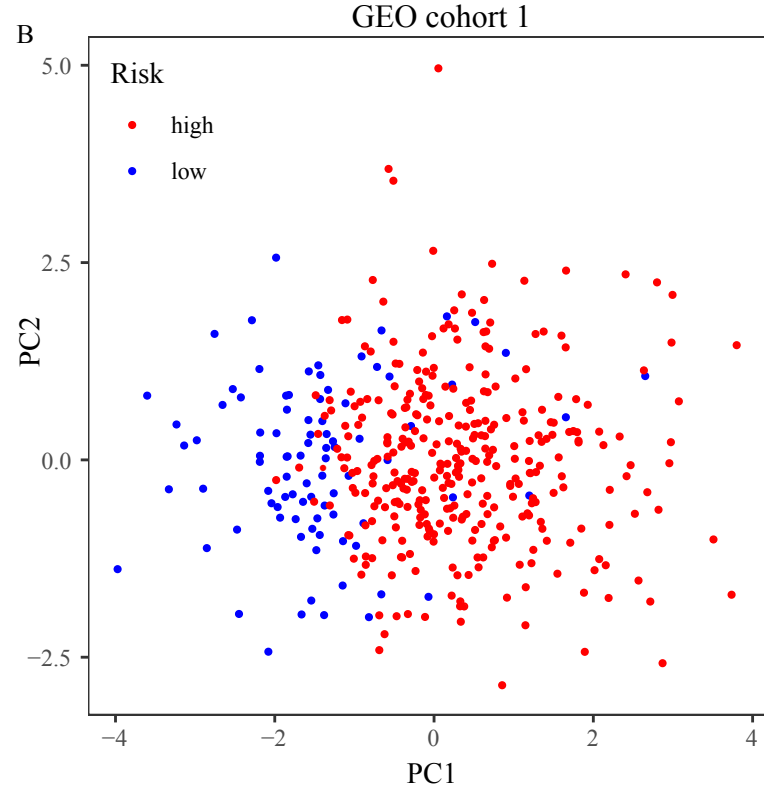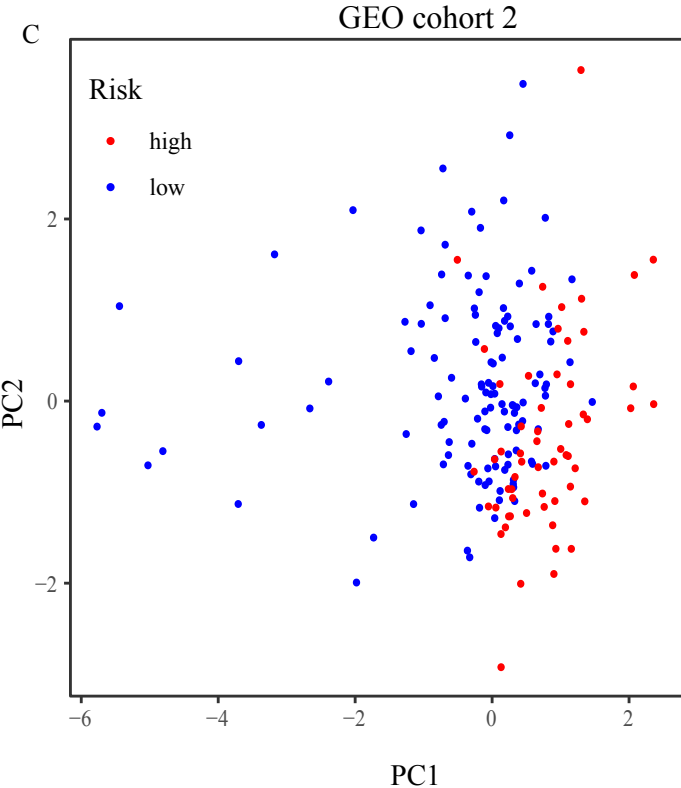

Supplement: Supplemental Information 1 — (A) Determination of the optimal cut-off risk score:1.515. (B) PCA showed that the patients in different risk groups were gathered in two areas in GEO cohort 1. (C) PCA showed that the patients in different risk groups were gathered in two areas in GEO cohort 2. [file peerj-09-11233-s001.pdf]

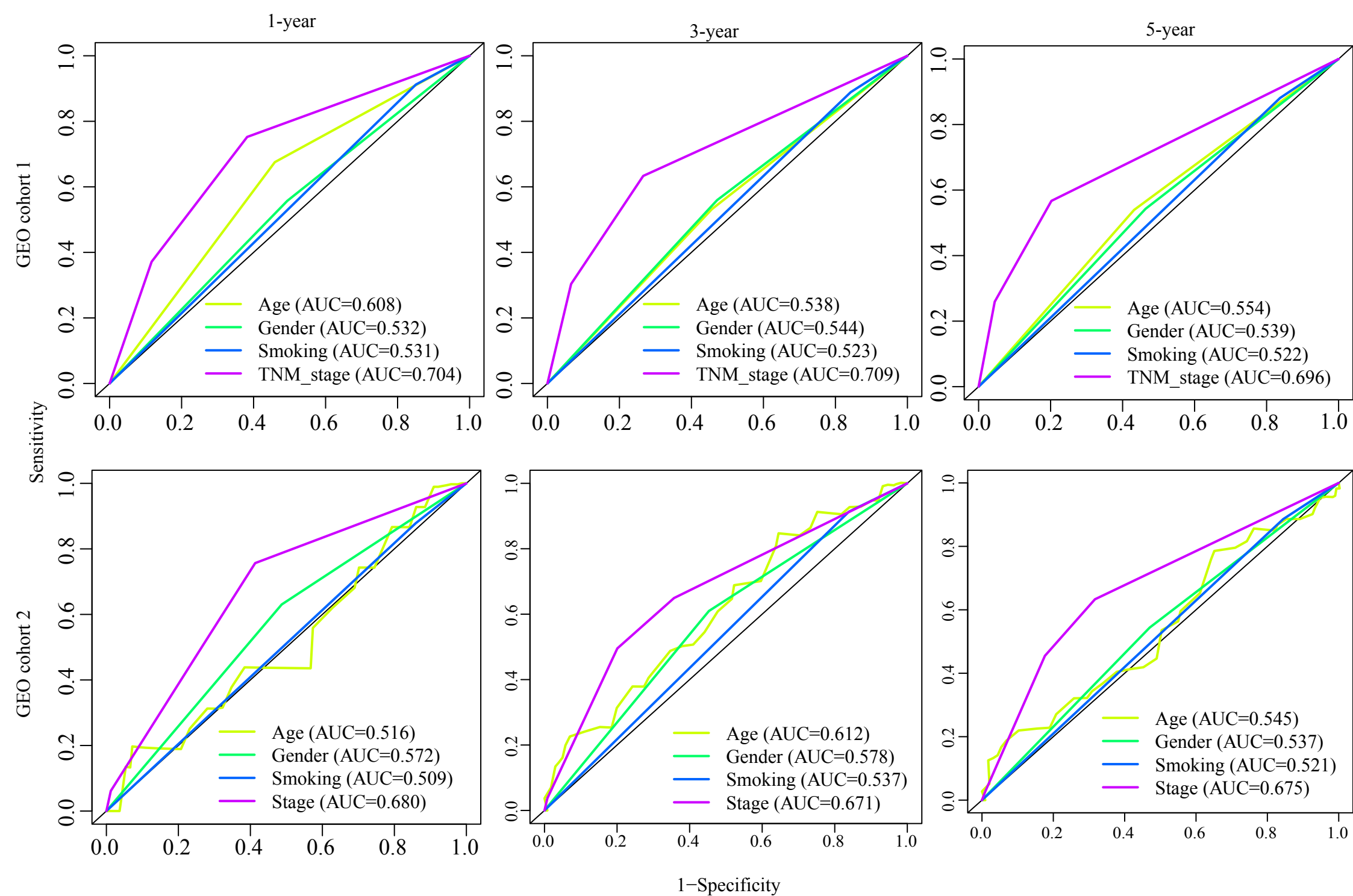

Supplement: Supplemental Information 2 [file peerj-09-11233-s002.pdf]

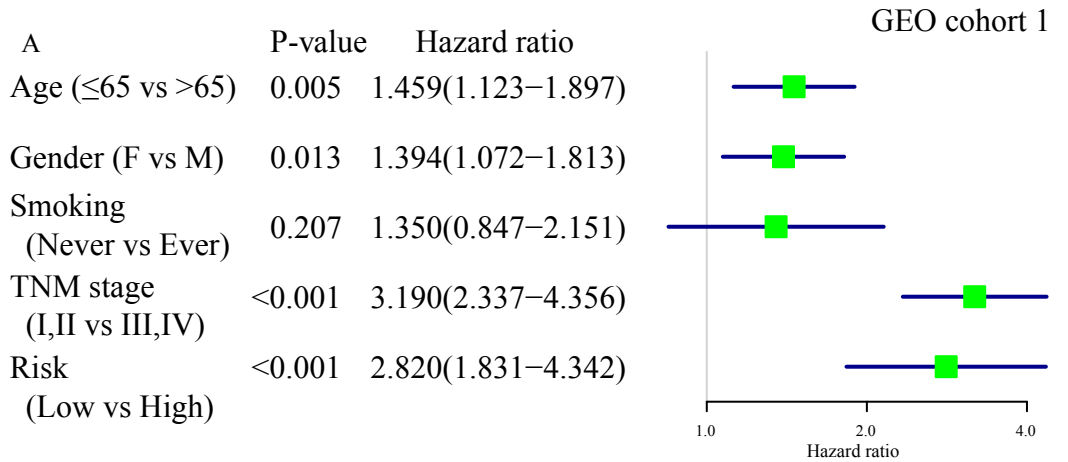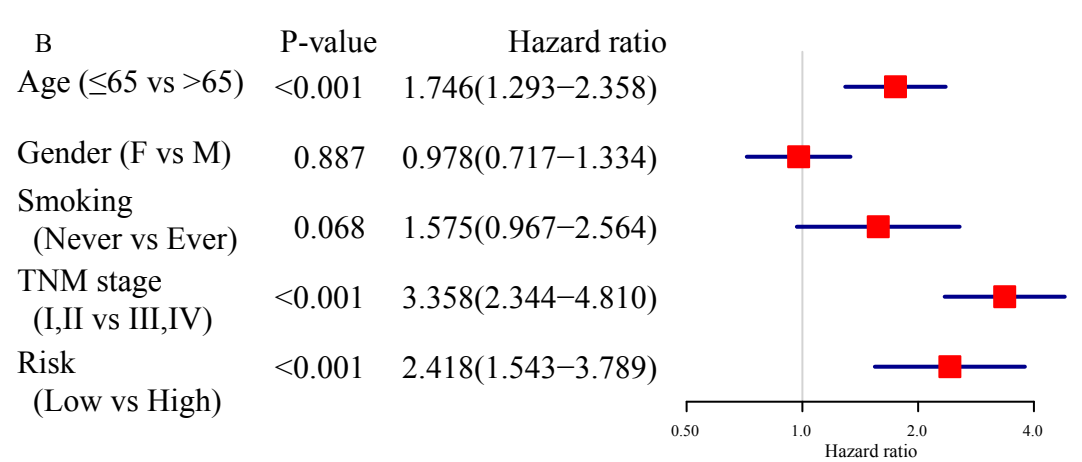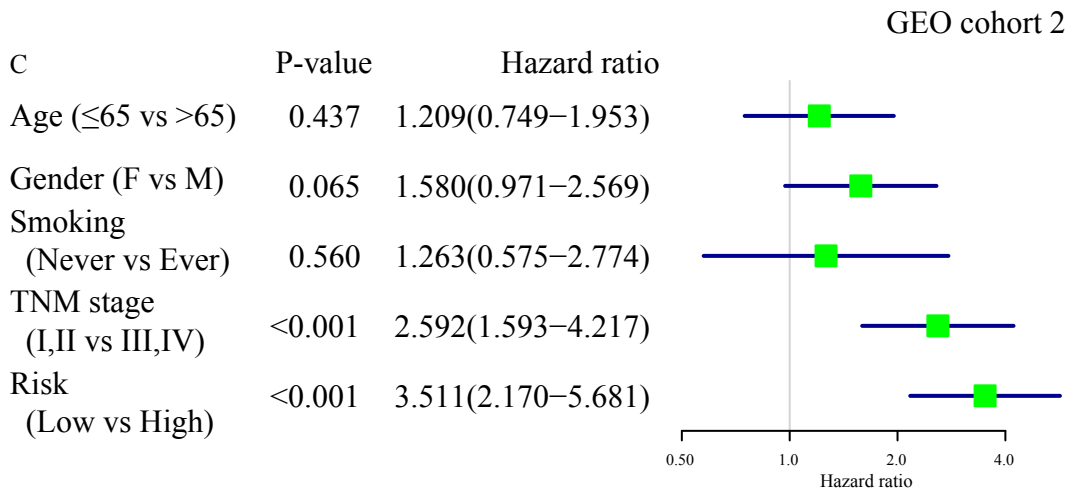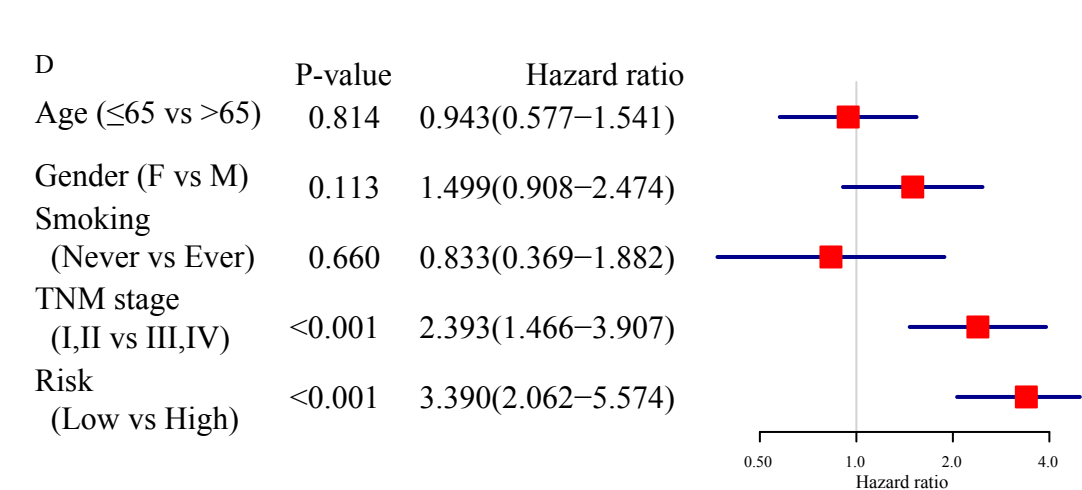

Supplement: Supplemental Information 3 — (A) Result of the univariate Cox regression analysis in GEO cohort 1. (B) Results of the multivariate Cox regression analysis in GEO cohort 1. (C) Result of the univariate Cox regression analysis in GEO cohort 2. (D) Results of the multivariate Cox regression analysis in GEO cohort 2. [file peerj-09-11233-s003.pdf]
